# Elucidation of Cellular Contributions to Heparin-Induced Thrombocytopenia Using Omic Approaches

**DOI:** 10.3389/fphar.2021.812830

**Published:** 2022-01-21

**Authors:** Jason B. Giles, Elise C. Miller, Heidi E. Steiner, Jason H. Karnes

**Affiliations:** ^1^ Department of Pharmacy Practice and Science, University of Arizona College of Pharmacy, Tucson, AZ, United States; ^2^ Department of Biomedical Informatics, Vanderbilt University Medical Center, Nashville, TN, United States

**Keywords:** heparin-induced thrombocytopenia, adverse drug reaction, t-cell, neutrophil, genomics, transcriptomics, metagenomic, proteomic

## Abstract

Heparin-induced thrombocytopenia (HIT) is an unpredictable, complex, immune-mediated adverse drug reaction associated with a high mortality. Despite decades of research into HIT, fundamental knowledge gaps persist regarding HIT likely due to the complex and unusual nature of the HIT immune response. Such knowledge gaps include the identity of a HIT immunogen, the intrinsic roles of various cell types and their interactions, and the molecular basis that distinguishes pathogenic and non-pathogenic PF4/heparin antibodies. While a key feature of HIT, thrombocytopenia, implicates platelets as a seminal cell fragment in HIT pathogenesis, strong evidence exists for critical roles of multiple cell types. The rise in omic technologies over the last decade has resulted in a number of agnostic, whole system approaches for biological research that may be especially informative for complex phenotypes. Applying multi-omics techniques to HIT has the potential to bring new insights into HIT pathophysiology and identify biomarkers with clinical utility. In this review, we review the clinical, immunological, and molecular features of HIT with emphasis on key cell types and their roles. We then address the applicability of several omic techniques underutilized in HIT, which have the potential to fill knowledge gaps related to HIT biology.

## Introduction

Heparin is a widely used anticoagulant indicated for a broad range of diseases and procedures. Approximately one third of all hospitalized patients in the United States receive heparin therapy (Campbell et al., 2000). This frequency of administration is due in part to multiple pharmacologic benefits of heparin, including immediate onset of action, rapid reversibility, and relative ease of titration (Bauer et al., 1997). However, these advantages are potentially offset by the immune-mediated complication, heparin-induced thrombocytopenia (HIT). HIT occurs in 0.2–2.7% of patients exposed to heparin anticoagulants and this risk increases in patients undergoing cardiac surgery (Warkentin et al., 1995; Warkentin et al., 2000; Girolami et al., 2003; Martel et al., 2005; Smythe et al., 2007). Despite the high prevalence and potentially life-threatening prognosis of this immune-mediated adverse drug reaction (ADR), the pathophysiology of HIT is still poorly understood making it difficult to predict and prevent.

HIT pathogenesis is initiated when heparin molecules, bound to circulating Platelet Factor 4 (PF4) proteins, are recognized by immunoglobulin G (IgG) antibodies (Kelton et al., 1994). These PF4/heparin antibodies then engage with platelets, leading to platelet activation and ultimately thrombocytopenia. Despite extensive research efforts towards understanding the immunopathology of HIT, fundamental knowledge gaps persist regarding HIT etiology (Cuker 2011; Greinacher et al., 2011; Karnes 2018). The HIT immunogen, the roles of antigen presenting cells and T-cells, and the B cell subtypes that produce the antibody remain unknown (Arepally 2017). The contributing role of non-platelet cell types in HIT, the interactions between cell types (platelets, monocytes, neutrophils, T-cells, etc.), and the heterogeneity of cell types intrinsic to HIT have yet to be fully resolved. Furthermore, the clinical significance of non-pathogenic PF4/heparin antibodies and the molecular basis that distinguishes them from pathogenic PF4/heparin antibodies remain unclear (Khandelwal and Arepally 2016). Transgenic mouse models (Zheng et al., 2015; Tutwiler et al., 2016; Perdomo et al., 2019), microfluidics devices (Tutwiler et al., 2016), and *in vitro* work on isolated cells (Kasthuri et al., 2012; Zhou et al., 2016) including platelets, peripheral blood mononuclear cells (PBMCs) (Kasthuri et al., 2012), and neutrophils (Duarte et al., 2019; Lelliott et al., 2020) have previously been employed to understand the pathophysiology of HIT. Although informative, prior targeted molecular approaches have not fully identified the mechanisms of HIT, likely due to the complicated and unusual nature of the HIT immune response. The rise in omics and “Big Data” over the last decade has resulted in a number of agnostic, whole system approaches for biological research that are especially informative for complex phenotypes. The common omics disciplines including genomics, transcriptomics, proteomics, metabolomics, and metagenomics, all have made great strides to answer questions in a wide range of biological topics. In the HIT field, however, these techniques have been under-utilized with studies employing only genome-wide association (Karnes et al., 2015; Karnes et al., 2017a; Witten et al., 2018) and array-based transcriptomic (Haile et al., 2017) approaches. This review will outline the current understanding of HIT pathogenesis within the context of specific cell types. This review will then evaluate omics techniques which might answer many unresolved questions related to HIT pathogenesis.

### Clinical Features of Heparin-Induced Thrombocytopenia

In contrast to most immune-mediated ADRs, the immune response in HIT is atypical and transient (Karnes et al., 2019). HIT is characterized by a fall in platelets (thrombocytes), 5–14 days after exposure to heparin (Prince and Wenham 2018). Mortality associated with HIT can reach 30% (Franchini 2005; Martel et al., 2005). PF4/heparin antibodies are necessary but not sufficient for HIT to occur, and these antibodies are typically produced 5–10 days after heparin exposure (Reilly et al., 2001; Staibano et al., 2017). IgG antibodies bind to PF4/heparin complexes to form ultra-large complexes (ULCs). These PF4/heparin antibodies are rarely detected in healthy individuals, with one study identifying ∼3% (Khandelwal and Arepally 2016) of the general population had detectable antibodies, using an optical density (OD) threshold of 0.4. A second study corroborated these findings and observed that 4.4% of healthy patients had antibodies against PF4/heparin (OD threshold = 0.5) (Krauel et al., 2011). In patients taking heparin, PF4/heparin antibodies are seen in 8–50% of patients (Arepally, 2017).

Up to half of patients with confirmed HIT experience thromboembolic complication including limb-threatening and life-threatening venous or arterial thrombosis (Prince and Wenham 2018). When thromboembolic events occur, the condition is sometimes referred to as HIT-associated thrombosis or HIT with thrombosis (HITT). In many patients, thromboembolic complications occur before a decrease in platelet count is observed (Prince and Wenham 2018). Less common presentations can also occur, such as skin necrosis and venous limb gangrene (Arepally 2017). Female sex, intravenous route of administration, and major surgery increase the risk of HIT (Arepally and Ortel 2010; Linkins et al., 2012). Patients receiving heparin within the last 90 days may experience rapid onset HIT within 24 h (Greinacher 2015). Patients receiving unfractionated heparin (UFH) are at higher risk of HIT compared to those receiving low molecular-weight heparin (LMWH) (Stein et al., 2009). One meta-analysis showed an absolute HIT risk of 0.2% with LMWH and 2.6% with UFH (Martel et al., 2005). Fondaparinux, a synthetic pentasaccharide fragment of heparin, shows almost no cross-reactivity with PF4/heparin antibodies and HIT is rare during fondaparinux treatment (Greinacher et al., 2017). Fondaparinux-associated HIT cases may be due to autoimmune HIT rather than fondaparinux. Autoimmune HIT occurs even in the absence of heparin but exhibits many clinical features of HIT (Greinacher et al., 2017). Proposed mechanisms of autoimmune HIT include endogenous polyanions, such as non-heparin glycosaminoglycans (GAGs), binding to PF4 complexes and exposing a neoepitope similar to heparin, which is subsequently recognized by IgG antibodies.

Confirmation of HIT requires both clinical and experimental confirmation. Clinically, a key indicator of HIT is an absolute drop in platelet count of 30–50% or a drop in platelet count of 40,000 to 80,000 per cubic mL (Greinacher 2015; Warkentin and Greinacher 2016). Platelet count along with other clinical indicators such as timing of onset of thrombocytopenia, presence of thrombosis or other sequelae, and alternative causes of thrombocytopenia collectively comprise the 4T scoring system (Warkentin and Greinacher 2016). Laboratory testing is also frequently used to confirm diagnosis of HIT. These laboratory tests include enzyme-linked immunosorbent assay (ELISA), which is used to test for PF4/heparin antibodies, and washed platelet activation assays such as the serotonin-release assay (SRA) and the heparin-induced platelet activation assay (HIPA) (Warkentin and Greinacher 2016), which quantify platelet activation in suspected patients’ serum. The SRA and HIPA assays are the “gold-standard” for detecting HIT but vary slightly in their assay endpoints. The SRA assay measures the release of radiolabel C^14^–serotonin from activated platelets while the HIPA test measures formation of platelet aggregation (Bakchoul et al., 2014). These functional assays are time-intensive and technically challenging, resulting in few laboratories having the regulatory and safety measures in place to perform these assays (Bakchoul et al., 2014). This limits their timeliness in clinical decision making and in confirming HIT cases.

### Immunological Features of Heparin-Induced Thrombocytopenia Pathogenesis

**FIGURE 1:**
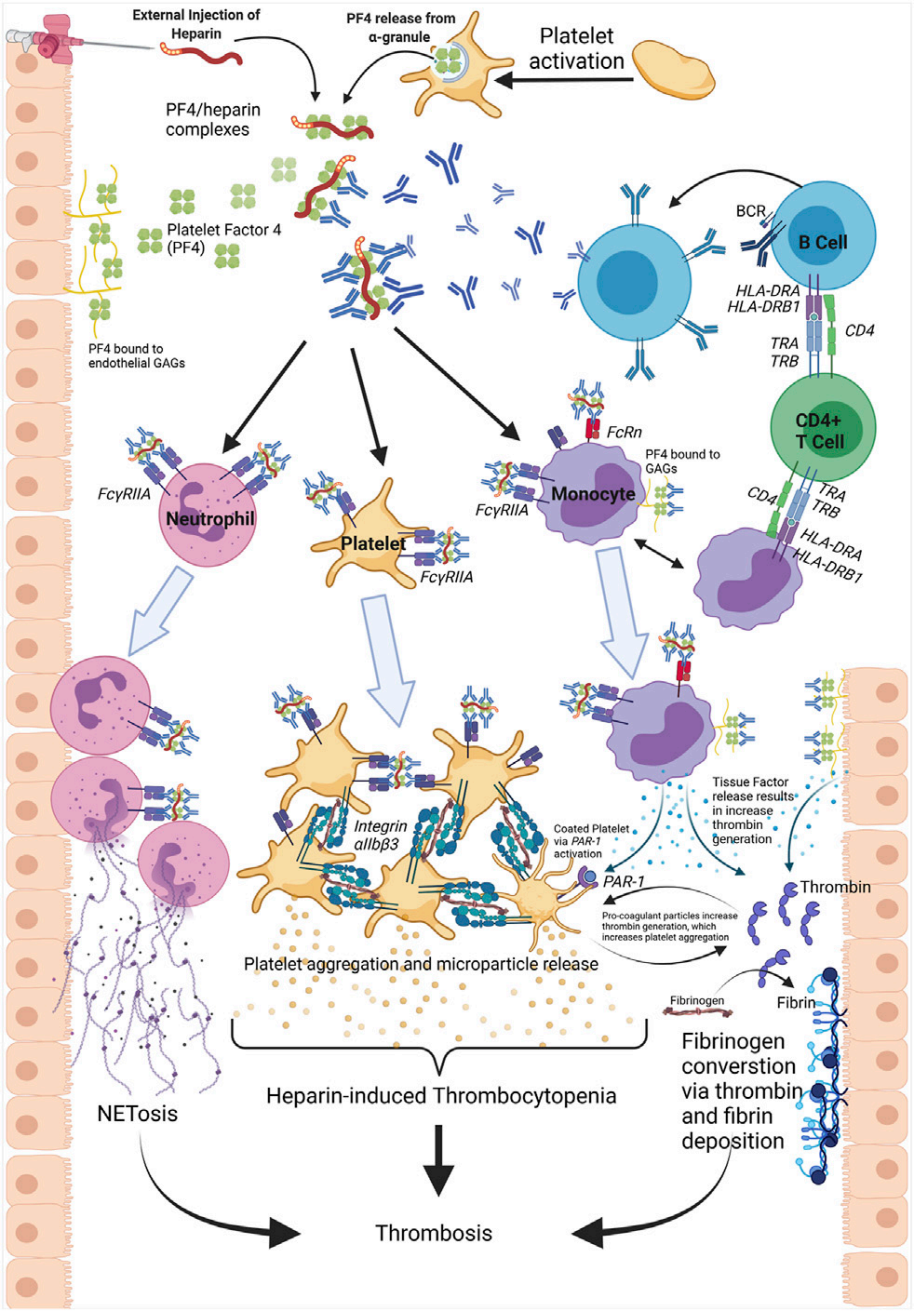
Heparin-induced thrombocytopenia (HIT) is initiated by the binding of heparin to platelet factor 4 (PF4), released from platelet *α*-granules. This binding results in a conformational change and exposure of a neo-epitope. PF4/heparin IgG antibodies generated from B cells bind to the neo-epitope, forming PF4/heparin/IgG ultra-large complexes (ULCs). The Fc region of the antibodies engage with FcγRIIa receptors on platelets, neutrophils, and monocytes. In platelets, this ULC binding results in intracellular activation, release of pro-coagulant microparticles, and subsequent platelet-platelet aggregation via Integrin *α*IIb/*β*3 engagement. The consumption of platelets into thrombi and platelet disintegration via microvesiculation causes thrombocytopenia. Neutrophil activation via ULC binding results in neutrophil recruitment to the endothelium and release of Neutrophil extracellular traps (NETs). The process of NETosis is a driver of thrombus formation and thrombosis. Monocyte activation leads to tissue factor (TF) expression and an increase in thrombin generation. Conversion of fibrinogen to fibrin via thrombin also contributes to an increased risk of thrombosis. TF released from monocytes additionally transactivates platelets via PAR-1 receptors, leaded to highly thrombotic, coated platelets. Furthermore, GAGs expressed on the endothelium act as binding partners for PF4 and subsequent antibody recognition. This antibody deposition results in increased TF release, thrombosis risk and serves as a site for activation of cells containing FcγRIIa receptors.

HIT is initiated by ULCs cross-linking with the Fc domain of PF4/heparin antibodies via the low affinity IgG Fc region receptor II-a (FcγRIIa) on the surface of platelets. This initiates platelet activation and release of procoagulant microparticles (Warkentin et al., 1994), culminating in thrombocytopenia and/or thrombotic complications (Rollin et al., 2015). ULCs have been shown to interact with monocytes (Arepally and Mayer 2001; Pouplard 2001; Kasthuri et al., 2012; Tutwiler et al., 2016), endothelial cells (Arepally and Mayer 2001; Arepally 2002; Arepally 2017), neutrophils (Perdomo et al., 2019), and platelets (Figure 1).

The immunopathogenesis of HIT is atypical and has characteristics of both innate and adaptive immunity (Greinacher 2015; Arepally 2017). Rapid production of PF4/heparin IgG antibodies, typical of a secondary adaptive immune or anamnestic response, is seen as early as 5 days after an initial heparin exposure. This response may be due to prior expose of PF4 bound to heparin-like GAGs or polyanions (Krauel et al., 2011; Staibano et al., 2017). However, PF4/heparin antibodies are transient, disappearing after approximately 90 days, characteristic of a non-anamnestic response (Warkentin and Kelton 2001). Heparin re-exposure after this 90 day period also does not reliably induce HIT, and patients with a previous HIT episode may be rechallenged with heparin (Warkentin and Sheppard 2014). PF4/heparin complexes activate complement, characteristic of an innate immune response. Binding of ULCs to B cells is mediated through the interaction between C3/C4 complement and complement receptor 2 (CR2 [CD21]). (Khandelwal et al., 2016). Polyreactive immunoglobulin M (IgM) antibodies initiate complement activation by binding to PF4/heparin complexes. IgM binding generates ULCs, facilitates the deposition of complement components, and promotes ULC binding to B cells (Khandelwal et al., 2018).

### Molecular Features of Heparin-Induced Thrombocytopenia Pathogenesis

Heparin is a naturally occurring, endogenous GAG, and it has been extensively studied in both the context of HIT and in its pharmacologic action. In HIT, heparin molecules bind to complexes of circulating PF4, a chemokine (chemokine C-X-C motif 4 [CXCL4]) that is stored in *α*-granules of platelets (Delcea and Greinacher 2016). Polyanionic heparin binds to positively charged PF4, but this must occur in specific molar ratios or charge imbalances and complex instability will prevent ULC formation (Suvarna et al., 2007). Studies have identified structural (Cai et al., 2016) and thermodynamic (Brandt et al., 2014; Kreimann et al., 2014) features necessary for formation of ULCs. While PF4 exists in monomer, dimer, and tetramer states, PF4 must exist in a tetrameric configuration for ULCs to form and HIT to occur (Mayo and Chen 1989; Cai et al., 2015). The tetrameric structure of PF4 displays a pseudosymmetry exhibiting an “open” and “closed” end characterized by distances between two salt bridge forming amino acids, glutamic acid 28 and lysine 50. In PF4 monomers A and C, amino acids E28 and K50 are ∼3 angstroms (Å) apart and form stable salt bridges. The same amino acids in monomers B and D are too far apart to form a salt bridge (∼8 Å) without a bond mediator (Cai et al., 2015). Heparin binds in the “closed” end of the tetramer and stabilizes the self-association of the PF4 tetramer (Cai et al., 2016). Binding of heparin results in a conformational change in the PF4 tetramer complex and stabilizes the “open” end of the tetramer, exposing the epitope recognized by PF4/heparin IgG antibodies (Cai et al., 2016).

Circulating PF4 interacts with other polyanions, including nucleic acids, liposaccharides on bacteria (Greinacher 2015), and cellular GAGs, but PF4 has a greater affinity for heparin than for other GAGs (Arepally 2017). The atomic weight and structure of GAGs dictates the binding strength to PF4. UFH is more likely to stabilize the intramolecular interactions within the PF4 tetramer compared to LMWHs (Cai et al., 2016). Fondaparinux, shows an even greater loss of stabilizing potential for the PF4 tetramer.

## Cellular Contributions to Heparin-Induced Thrombocytopenia Pathogenesis

Platelets are a primary driver of HIT and platelet activation contributes to HIT’s thromboembolic consequences. While the role of platelets and megakaryocytes have been extensively studied, other cells play critical roles in HIT pathogenesis, including T-cells, monocytes, endothelial cells, and neutrophils (Table 1) (Arepally and Mayer 2001; Pouplard 2001; Xia and Kao 2002; Tutwiler et al., 2016) (Cines et al., 1987; Arepally 2002) (Zheng et al., 2015) (Perdomo et al., 2019).

**TABLE 1:** Mechanisms of PF4/heparin antibody binding, involvement in pathogenesis of heparin-induced thrombocytopenia, and involvement in HIT-associated thrombosis of various cell types.

Cell Type	PF4/heparin antibody binding	Involvement in HIT Pathophysiology	Involvement in HIT-Associated Thrombosis
Megakaryocytes (Platelets)	Direct: via FcγRIIa receptor	• PF4/heparin antibodies bind to platelets	• Activated platelets release microparticles with procoagulant activity Warkentin et al. (1994)
		• Activation leads to pro-coagulant particle release Warkentin et al. (1994)	• platelet-NETs lead to thrombus formation Martinod and Wagner (2014)
		• Apoptotic and non-Apoptotic depletion of platelets occur; leads to thrombocytopenia Mordakhanova et al. (2020)	• PF4-VWF-IgG on ECs leads to platelet deposition and thrombi enlargement Johnston et al. (2020)
		• TULA-2 deficiency increases thrombocytopenia Zhou et al. (2016)	• FcγRIIa 131 R R allele leads to increased thrombosis Rollin et al. (2015)
T-cells	Precursor: No direct evidence for PF4/heparin antibody mediated activation	• CD4 T-cells are necessary for antibody response Zheng et al. (2015)	• Lack of evidence for T-cells influence on thrombosis
		• Antigen presenting cells associated with PF4/heparin antibodies Karnes et al. (2017b), Zhang et al. (2019)	
		• Lack of memory B cells and PF4/heparin complex similarities to T-cell independent antigen confounds T-cells role Warkentin and Kelton (2001)	
Neutrophils	Direct: via FcγRIIa receptor	• Activation leads phagocytosis, degranulation, and NETs generation, which contain prothrombotic components Duarte et al. (2019)	• NETs are critical for thrombus formation exhibiting engagement with platelets, red blood cells and procoagulant proteins Perdomo et al. (2019)
			• L-selectin and CD11b/CD18 upregulation promote adhesion of neutrophil-platelet aggregation Xiao et al. (2008)
Monocytes	Direct: via FcγRIIa receptor and neonatal Fc receptor	• Activation of monocytes triggers TF expression and release of TF particles activates platelets Kasthuri et al. (2012)	• Binding of ULCs increase procoagulant activity indicated by release of TF containing particles
			• TF containing microparticles promote thrombin activation
			• PF4 bound to cell-surface GAGs serve as reservoirs for PF4/heparin antibody binding Rauova et al. (2010), Tutwiler et al. (2016)
Endothelial	Indirect: EC bound ULCs mediate activation of other cell types	• Source for TF release and site for PF4 deposition onto GAGs, subsequent binding of antibodies and engagement to FC-receptor on platelets Madeeva et al. (2016)	• Platelet adhesion to EC exacerbates thrombus formation and promotes thrombosis
			• EC activation releases VWF strings that bind PF4 and serve as new antigenic sites Hayes et al. (2017)
			• ECs, in the presence of platelets upregulate adhesion markers–E-selectin, ICAM-1 and VCAM Herbert et al. (1998)

EC indicates endothelial cells; FcγRIIa, low affinity immunoglobulin gamma Fc region receptor II-a; GAG, glycosaminoglycans; ICAM-1, intracellular adhesion molecule 1; NET, neutrophil extracellular traps; PF4-VWF-IgG, platelet factor 4–von Willebrand factor–immunoglobulin G complexes; TULA-2, T-Cell Ubiquitin Ligand-2; TF, tissue factor; ULC, ultra-large complexes; VCAM, vascular cell adhesion molecule; VWF, von Willebrand factor

### Platelets (Megakaryocytes)

Platelets, the circulating anuclear fragments derived from megakaryocytes, express a single class of Fcγ receptors, FcγRIIa (Gratacap et al., 1998). Binding of ULCs to the FcγRIIa receptor triggers intracellular signaling involving the spleen tyrosine kinase 50 (SYK50) (Arepally 2017). SYK is phosphorylated when bound to FcγRIIa, activating SYK and triggering downstream signaling through calcium mobilization and PI3-kinase activation (Zhou et al., 2016). This leads to integrin *α*IIb/*β*3 activation and release of procoagulant microparticles from platelet granules. Two types of granules are released from activated platelets: dense granules, containing serotonin, calcium, adenosine triphosphate, and adenosine diphosphate and *α*-granules containing PF4, P-selectin, and fibrinogen (Fitch-Tewfik and Flaumenhaft 2013; Arepally and Padmanabhan 2020; Tardy et al., 2020). As shown in Figure 1, this signaling cascade leads to platelet activation and aggregation upon fibrinogen binding to the integrin receptor (Gratacap et al., 1998), resulting in a positive feedback loop facilitating a hypercoagulant state.

While the mechanisms of platelet activation via the FcγRIIa receptor are well understood, the mechanism of thrombocytopenia in HIT is not fully resolved. Clearance of antibody-coated platelets in the spleen (Prince and Wenham 2018), the consumption of platelets by thrombi, and platelet disintegration through platelet-derived microvesicles are proposed mechanisms for thrombocytopenia in HIT (Liu et al., 2013; Liu et al., 2016). A recent study showed that platelet activation was accompanied by cell death through complex apoptotic and non-apoptotic pathways, which may contribute to thrombocytopenia (Mordakhanova et al., 2020). Platelet activation and aggregation into thrombi reduce platelet count (Greinacher 2015). More recently, von Willebrand factor (VWF) and complexes of PF4-VWF-IgG antibodies were shown to bind extensively to platelets (Chen and Chung 2018) (Johnston et al., 2020).

Mutations in the FcγRIIa receptor have been shown to be associated with both HIT and HITT (Karnes 2018). The polymorphism H131R in the IgG binding region of FcγRIIa receptor has been associated with HIT in several populations, but a meta-analysis showed no difference in HIT between the wild-type and 131R variant (Trikalinos et al., 2001). However, individuals homozygous for the 131-RR genotype were at higher risk of HITT, likely due to the inability of endogenous IgG2 antibodies to bind to the RR genotype receptor. IgG2 antibodies compete with PF4/heparin antibodies (IgG1) for binding to FcγRIIa (Rollin et al., 2015). Decreased IgG2 affinity allows increased engagement of the IgG1 antibody, further stimulating the intracellular pathway involving FcγRIIa, SYK50 and platelet activation.

### T-Cells

After maturation, selection, and transport out of the thymus, subsets of T-cells including regulatory T-cells (Treg), naïve T-cells, and memory T-cells are critically important for the maintenance of immune response and immunological memory. T-cells, particularly those subsets involved in antigen presentation, have been implicated in the immune response seen in HIT patients. HIT exhibits T-cell dependent characteristics (Xia and Kao 2002, Xia and Kao 2003; Chudasama et al., 2010; Joglekar et al., 2015), such as the requirement of CD4 and CD40 ligands on T-cells for PF4/heparin IgG production (Zheng et al., 2015). Observed associations between Human Leukocyte Antigen (HLA-DR) alleles and HIT suggest T-cell dependent mechanisms (Karnes et al., 2015; Karnes et al., 2017b; Zhang et al., 2019). However, HIT also presents T-cell independent characteristics. PF4/heparin complexes have similar characteristics to T-cell independent antigens, including similar molecular weight, repetitive epitopes, and spacing between elements (Vos et al., 2000; Staibano et al., 2017). The lack of memory B cells after heparin exposure (Selleng et al., 2010), and the role for marginal B cells in PF4/heparin IgG production in mouse models (Jouni et al., 2016) point to T-cell independent mechanisms.

### Neutrophils

Neutrophils are phagocytes and the most abundant type of white blood cell in the body (Van Rees et al., 2016). They migrate through blood vessels and perform a variety of function such as recruitment to sites of inflammation, phagocytosis of pathogens, degranulation, and generation of neutrophil extracellular traps (NETs). Activation of neutrophils via FcγRIIa receptor promotes phagocytosis, degranulation, and production of reactive oxygen species (ROS) (Gollomp et al., 2018). Neutrophils exposed to PF4 and PF4/heparin antibodies show enhanced adhesion to venous endothelium. In a microfluidics model, enhanced neutrophil involvement within venular thrombi was observed after induction of PF4 and PF4/heparin antibodies (Gollomp et al., 2018). Changes in absolute neutrophil counts have also been observed in HIT patients (Hui et al., 2019).

Neutrophils may play a role in HIT through generation of neutrophil extracellular traps (NETs). NETs are complexes of intracellular components including histones, myeloperoxidase, and elastase. NETs, when released from neutrophils, bind to pathogens and have anti-microbial activity (Van Rees et al., 2016). NETs have also been shown to contain prothrombotic components, such as tissue factor (TF), factor XII, VWF, and fibrinogen (Perdomo et al., 2019). Increased levels of cell-free DNA (cf-DNA), myeloperoxidase (MPO), elastase, VWF, and citrullinated histone H3 (CitH3), which are all markers of NETosis, have been reported in HIT patient plasma (Perdomo et al., 2019). In one study, whole blood from HIT patients included a second population of neutrophils that were not present in healthy controls, classified as activated neutrophils or low-density granulocytes (LDGs) (Perdomo et al., 2019). Using microfluidics models, complexes of NET and PF4 were shown to be recognized by KKO antibodies, which are synthetic antibodies that mimic PF4/heparin antibodies, and IgG from the plasma of HIT patients (Gollomp et al., 2018). Recently, murine HIT models (FcγRIIa+/hPF4+) recapitulated *ex vivo* results, in which NETosis was required for thrombus formation. Inhibition of NET formation by the NET inhibitor, GSK484, caused a marked reduction in thrombus deposition and neutralization of FcγRIIa completely abolished thrombi. However, blocking of platelet-neutrophil interactions via anti-CD62p did not inhibit thrombus formation (Perdomo et al., 2019).

More recent studies show neutrophils and the release of NETs do not initiate thrombosis in HIT mouse models, but play a role in thrombus growth and stabilization. Loss of peptidyl arginine deiminase type IV (PAD4) abolishes neutrophils’ ability to release NETs. Pad4 −/− HIT mice following KKO injection had thrombocytopenia comparable to Pad4 +/+ HIT mice. However, loss of PAD4 led to smaller venular thrombi following injury but did not abrogate thrombosis all together (Gollomp et al., 2018). Other studies have shown that heparin-induced NETs display two markedly different phenotypes. The first NETs (described above) are from neutrophils where cell nuclear membranes are disrupted, and the second subset are from neutrophils where small amounts of extracellular DNA were released but those neutrophils maintained their structural integrity (Lelliott et al., 2020). One study showed neutrophil activation induced by ULCs varied among individuals. In fact, an individual’s neutrophil response to ULCs remained fixed over a longitudinal 1-year period (Duarte et al., 2019), suggesting that susceptibility to neutrophil activation by ULCs is specific to the host and may be genetic in nature. These studies indicate that NETosis is a driver of thrombosis in HIT, but inter-individual variability in neutrophil count and heterogeneity, including polymorphism in cell surface receptors, are potential modifiers of HITT risk.

### Monocytes

Monocytes, the immature precursor to macrophages and dendritic cells, serve as vehicles for antigen presentation, cytokine production, tissue remodeling, and phagocytosis (Guilliams et al., 2018). Monocytes express GAGs that react with PF4 to generate structural changes in PF4, which allows binding of PF4/heparin antibodies (Rauova et al., 2009). Monocytes primarily express two GAGs, heparin sulfate (HS) and dermatan sulfate (DS) (Madeeva et al., 2016). These GAGs may be the preferred target for PF4 binding and formation of ULCs, compared to platelets, as platelets express a low affinity GAG, chondroitin sulfate (Rauova et al., 2010). The binding of PF4/heparin antibodies to these PF4/GAG complexes on monocytes results in activation of Fc-receptors on monocytes.

Binding of PF4/heparin antibodies to FcγRIIa receptors trigger monocyte TF expression and release of TF particles (Kasthuri et al., 2012). TF binds to Factor VII (FVII)/FVIIa complexes and initiates coagulation by activating FX and FIX. The conversion of FX to FXa in complex with FVa activates prothrombin to thrombin (Mackman, 2004) (Tutwiler et al., 2016). The removal of monocytes from whole blood decreases platelet accretion and fibrin deposition. When levels of monocytes are brought back to normal physiological levels, the ability of KKO antibodies to stimulate platelet accretion and fibrin deposition is restored (Madeeva et al., 2016). Recent work has shown that in HIT patients, absolute monocyte levels decrease over a 7-days span post-HIT (Hui et al., 2019). However, overall counts of monocytes did not vary between patients with HIT and HITT (Hui et al., 2019).

The activation of monocytes and release of TF has recently been shown to involve the neonatal Fc receptor (FcRn) (Cines et al., 2020). Dual receptor engagement of ULCs (FcγRIIa and FcRn) on monocytes may explain why KKO antibodies have higher binding affinity to monocytes compared to platelets (Rauova et al., 2010). Thrombin generated by stimulated monocytes can *trans*-activate platelets via protease-activated receptor 1 (PAR-1), a subfamily of G protein-coupled receptors (Tutwiler et al., 2016). Coactivation of platelets via PAR and FcγRIIa leads to the formation of highly prothrombotic, coated platelets (Figure 1) (Tutwiler et al., 2016). Following whole blood exposure to KKO and PF4, a population of platelets exist with increase expression of P-selectin and other markers of coated platelets (Tutwiler et al., 2016). Expression of coated platelet markers was considerably reduced in the absence of monocytes in mouse platelet-rich plasma and in monocyte-depleted whole blood (Tutwiler et al., 2016). The amount of PF4 deposition onto monocytes is likely correlated with the extent of platelet transactivation (Madeeva et al., 2016). This amplification loop where activated platelets release PF4, this PF4 binds to monocytes, resulting in release of TF particles, which then further activates platelets and generates coated platelets, illustrates the complex interplay of cells in HIT.

### Endothelial Cells

Endothelial cells line blood vessels and play a critical role in regulating blood flow, vascular homeostasis, vascular tone, and platelet function (Jain et al., 2016; Madeeva et al., 2016; Sturtzel 2017). Signaling between vascular endothelial cells and platelets is vital for regulation of thrombosis (Jain et al., 2016). The basal state of endothelium is anticoagulant in nature, but at the onset of tissue trauma the endothelium facilitates coagulation (Sturtzel 2017). The endothelium expresses a mosaic sheath of glycoproteins, proteoglycans, and GAGs, known as the glycocalyx, under normal physiological conditions (Madeeva et al., 2016). Endothelial cells may bind PF4 with a greater affinity than platelets or monocytes given the predominant GAG on endothelial cells is heparin sulfate (Madeeva et al., 2016).

As shown in Figure 1, platelet activation at the site of injured endothelium releases PF4, which is deposited onto the endothelium due to a high affinity for GAGs. The PF4/GAG complex then becomes a target for PF4/heparin antibody binding. This binding contributes to increased endothelial activation and recruitment of platelets in a feed forward loop resulting in fibrin deposition and thrombosis (Madeeva et al., 2016). PF4 release was associated with binding of ULCs on thrombi following laser injury of endothelial cells (Hayes et al., 2017). Complexes of PF4 and VWF, released from endothelium following injury, may also be an antigenic site for PF4/heparin antibodies (Johnston et al., 2020). Microfluidics injury models also support that endothelial cells are the main initial harbor for PF4 released from activated platelets and a target for PF4/heparin antibodies (Hayes et al., 2017). The higher affinity of endothelial GAGs for PF4 may help to explain why antiplatelet therapy is not efficacious in HIT (Madeeva et al., 2016) and why thrombotic conditions may persist after heparin is no longer present. As approaches to date have yet to resolve these questions, applying multi-omics techniques to HIT has the potential to bring new insights and fundamentally improve our understanding.

## Application of Omic Approaches to Heparin-Induced Thrombocytopenia

Application and integration of omics disciplines, such as genomics, transcriptomics, metagenomics, proteomics, and metabolomics, has the potential to probe cellular roles discussed above and reveal previously unknown aspects of HIT biology. The complex, immune-mediated nature of HIT makes it well-suited for multi-omics approaches to solve questions surrounding the pathogenesis of HIT.

### Genomics

The majority of genetic studies related to HIT have been candidate gene studies. (Arepally et al., 1997; Pouplard et al., 2012; Rollin et al., 2012; Rollin et al., 2013; Rollin et al., 2015; Karnes et al., 2017b; Zhang et al., 2019). These studies focus on genes or single nucleotide polymorphisms (SNPs) with suspected biological relevance to the disease (Tabor et al., 2002). Genomic association studies for HIT and HITT are summarized in Table 2. Such studies have identified multiple associations with HIT and HITT (Brandt et al., 1995; Arepally et al., 1997; Rollin et al., 2015). Candidate gene/SNP studies can be cost effective, but these approaches have multiple limitations. They include the need for *a priori* information on gene function and a historical inability to replicate observed associations (Tabor et al., 2002; Patnala et al., 2013). Genome-wide association studies (GWAS) are an “agnostic” approach, in contrast to the hypothesis-driven candidate gene studies, and have become a powerful tool in discovering the genetic influence of complex disease. GWAS investigates the association between millions of genetic variants and a phenotype of interest. Despite clear successes in GWAS in identifying genetic polymorphisms associated with other phenotypes/diseases, very few GWAS studies have been performed for HIT (Karnes et al., 2015; Witten et al., 2018). One study identified a SNP in chromosome 5 in AC106799.2 as a risk allele for HIT (Witten et al., 2018). This GWAS only contained 96 suspected HIT cases and a replication cohort of 86 suspected HIT cases. Another GWAS observed a significant association with HIT near TDAG8 (or GPR65) (Karnes et al., 2015). Low sample sizes, particularly in HIT cases, were a limitation in both studies and reduce the impact of these findings. Furthermore, many of the HIT cases in both studies were determined through antibody testing and 4Ts scores and HIT was not confirmed with functional assays. Additionally, discovery cohorts in both studies did not include differentiation between antibody positive patients who develop HIT and antibody positive patients who did not develop HIT. This creates ambiguity in these results as the associations seen may be with PF4/heparin antibody levels and not HIT itself.

**TABLE 2:** Genomic studies identifying associations with Heparin-Induced Thrombocytopenia.

Locus	Variant(s)	Phenotype [Assay for Confirmation]	Study Design	Study Limitations–Pathogenesis Evidence	Citation
*TDAG8 (or GPR65)*	*rs1887289 rs10782473*	*HIT, Antibody production [ELISA]*	*GWAS*	Small cohort (*n* = 67 HIT, 884 controls); no functional assay; replication of signal in antibody production but not HIT–Signal near TDAG8 gene (T-cell death-associate gene 8) may be involved in antibody response not HIT itself; additional studies/larger cohorts needed for confirmation of gene influence	Karnes et al. (2015)
*Chr 5 near AC106799.2*	*rs1433265*	*HIT [ELISA or HIPA]*	*GWAS*	Small discovery (*n* = 96 HIT, 96 controls) and replication (*n* = 86 HIT, 86 controls) cohort; signal did not reach GWAS significance threshold–SNP identified is located in non-coding gene region; influence on HIT requires further (*in vitro/in vivo*) validation	Witten et al. (2018)
*IL-10*	*IL10G G20 microsatellite*	*PF4/heparin antibody production [ELISA and SRA]*	*Candidate Gene*	No replication: small cohort (82 HIT 84 Ab^pos^, 85 Ab^neg^)–IL-10 plays critical role in peripheral inflammation; marker of immune response, may not be specific to antibody production in HIT	Pouplard et al. (2012)
*FCGR2A*	*rs1801274 [H131R]*	*HITT [ELISA and SRA]*	*Candidate Gene*	Small cohort (*n* = 35 HITT, 54 HIT, 160 Ab^pos^, 174 Ab^neg^, 206 healthy controls)–Strong evidence for direct influence of polymorphism on phenotype (thrombosis); Amino acid substitution located in extracellular domain of FcγRIIa; interacts with Fc fragment of IgG antibody; multiple other studies have observed same association Burgess et al. (1995), Carlsson et al. (1998)	Rollin et al. (2015)
*HLA-DR*	DRB3*01:01	*HIT [ELISA]*	*Candidate Gene*	Small cohort (*n* = 65 HIT, 350 controls); no functional assay for HIT confirmation; no replication–Lack of evidence for presentation of HIT antigen via HLA using traditional (*in vitro/in vivo*) techniques; lessens study impact of HLA allele as risk factor for HIT	Karnes et al. (2017b)
*HLA-DR*	DRB1*03:01 and DQB1*02:01	*Antibody production [ELISA]*	*Candidate Gene*	No replication: no functional assay–Haplotype has been associated with other autoimmune disorders; lack of evidence for presentation of HIT antigen via HLA using traditional (*in vitro/in vivo*) techniques lessens impact of findings	Zhang et al. (2019)
*ACP1*	*rs11553742/rs11553746 haplotypes*	*Antibody production [ELISA and SRA]*	*Candidate Gene*	No replication: no difference in genotype frequency between Ab^pos^ and HIT patients, solely Ab^neg^ vs Ab^pos^–Evidence does exist for influence in antibody response as ACP1 regulates ZAP-70, which plays critical role in T-cell development; functional studies necessary to support findings	Rollin et al. (2013)
*PTPRJ*	*rs1566734*	*HIT [ELISA and SRA]*	*Candidate Gene*	No replication: All patients (Ab^neg^, Ab^pos^, HIT) were cardiopulmonary bypass patients; potential enrichment of population–Influence on phenotype supported by amino acid changing variants identified; previous murine studies strengthen support Senis et al. (2009), Ellison et al. (2010)	Rollin et al. (2012)
	*rs1503185*				

Ab^neg^: negative PF4/antibody assay; Ab^pos^: positive PF4/antibody assay with negative functional assay result; GWAS: genome-wide association study; HIPA: heparin-induced platelet aggregation assay; SRA: serotonin release assay, HIT: heparin-induced thrombocytopenia; HITT: heparin-induced thrombocytopenia associated thrombosis; Chr, chromosome.

Within the HIT field, there is a need for a well-powered GWAS study which can overcome the limitations of the previous literature. Applying GWAS strategies to HIT research could have its largest impact in 1) identifying novel variants associated with HIT and 2) corroborating previous findings of variants which alter HIT risk. Large GWAS could provide valuable information on additional polymorphisms that alter thrombosis risk in HIT. GWAS could be used to identify genetic variants associated with antibody production independent of HIT and bring new insights into why only a subset of antibody positive patients develop HIT. However, the acquisition of enough HIT cases to create a sufficiently powered GWAS for HIT is a challenge. Whole-genome sequencing (WGS) might also be used to discover new polymorphisms with an influence on HIT. Declining sequencing costs will permit large scale interrogation of entire genomes, including rare variants, in the near future (Tam et al., 2019). However, WGS approaches will likely require even greater sample sizes for statistical power and additional resources for data storage, processing, and analysis.

### Metagenomics

Microorganisms, present throughout the human body, including bacteria, viruses, and fungi, collectively defined as the human microbiota, may have more biological influence that previously expected. Metagenomics, or the surveying of all (“Meta-”) microbial genomes (genomics), is typically performed via shotgun or 16s rRNA sequencing. Briefly, 16s rRNA sequencing is a cost-effective solution to surveying the communities of a bacterial microbiome via a single gene, the 16S ribosomal gene. Whereas shotgun sequencing surveys the entire genome of all organism present in a sample, including human, viral and fungal DNA (Ranjan et al., 2016). Recent evidence suggests that HIT is a misguided immune response, involving a prior bacterial exposure serving as a “priming” event. PF4/polyanion antibodies are produced and later recognize heparin as a “foreign” entity. PF4 has been shown to interact with both gram-positive and gram-negative bacteria and antibodies obtained from HIT patients recognize PF4 bound to bacteria (Krauel et al., 2011). Additional studies show PF4/heparin antibodies present in a cohort of subjects with periodontal disease (Greinacher et al., 2011) as well as inpatients with bacteremia. Patients with gram-negative bacteria showed higher levels of PF4/heparin antibodies relative to healthy controls (Pongas et al., 2013). Mice with polymicrobial sepsis produced PF4/heparin antibodies which followed the expected seroconversion from IgM to IgG (Jouni et al., 2016). The rapid antibody class switching observed in HIT, may in fact be due to a prior exposure of PF4 bound to polyanions expressed on bacteria or those with ongoing infections (Pongas et al., 2013). This evidence suggests the response against PF4/heparin complexes may be a misguided immune reaction, in that the epitope generated by PF4/heparin “mimics” the epitope of PF4 when bound to bacteria cell wall components. As this theory of a misguided immune response in HIT has yet to identify specific classes or even phyla of bacteria, sequencing the microbiome may provide powerful insights into HIT pathogenesis.

Sequencing of microbiomes from antibody positive patients compared with that of antibody negative controls could elucidate if the presence of particular microorganisms is associated with detectable PF4/heparin antibodies. Patients with specific bacterial exposure, identified through presence of bacteria or relevant antibodies, could alert clinicians to the increased risk of HIT prior to heparin exposure. Studies have already observed that gut microbiota alter the risk of cardiovascular disease (Witkowski et al., 2020; Jin et al., 2021). Gut microbiota-derived metabolites may increase the risk of venous thromboembolisms (Zhu et al., 2016; Hasan et al., 2020; Lichota et al., 2020). However, the influence of gut microbiota-derived metabolites has not been evaluated in the context of HIT or HITT. Given the number of patients prescribe heparin relative to the few who develop HIT, there are methodological difficulties in the assessment of all heparin-exposed patients prior heparin administration.

### Transcriptomics

Transcriptomics, the comprehensive study of all RNA transcripts within an organism, is a powerful approach to explore cellular functions. The dominant techniques employed today are microarrays and RNA-sequencing (RNA-seq). Microarrays measure the abundance of a predefined set of transcripts and can allow the assay of thousands of transcripts simultaneously. Conversely, RNA-seq quantifies the entire transcriptome of cells via the sequencing of cDNA transcripts. Numerous HIT studies have looked at targeted mRNA levels using quantitative real-time PCR. Often these studies focus on a specific gene of interest (Lhermusier et al., 2011; Kasthuri et al., 2012; Rollin et al., 2015) as opposed to the broader scope that microarrays provide. Microarray studies could identify new roles of mRNA transcripts and their genes involved in HIT. This information could be used to delineate individuals who may produce higher antibody quantities and increased monitoring of those patients could be implemented to help minimize the progression to thromboembolic complications of HIT. Microarrays require a priori information on specific transcripts, but RNA-Seq provides a more agnostic approach for surveying the transcriptome. RNA-seq, most often utilized for differential gene expression (DGE), allows users to determine quantitative changes in expression levels between experiments, cases/controls studies, different disease states, etc.

Transcriptomics work could help identify specific transcriptional activity present in HIT cases compared to non-HIT controls in cross sectional studies. Also informative may be longitudinal studies on patients during HIT and post-HIT after antibodies have waned to interrogate the transcriptional differences where patients can serve as their own controls. Despite the clear applicability of RNA-seq in investigating HIT cases/controls, or even antibody cases/controls, RNA-seq has not yet been applied to HIT research. The most common RNA-sequencing technology, short read cDNA sequencing for DGE, is a robust and highly reproducible across and between platforms (Stark et al., 2019). Applying RNA-seq to HIT research might have a large impact in determining novel biological pathways involved in HIT pathogenesis. This application might also identify differentially expressed genes responsible for the subset of PF4/heparin antibody positive patients who go on to develop full-blown HIT. RNA-seq has the potential to discovery if difference in transcript levels amplify, or suppress, the antibody signally cascade initiated by IgG-platelet binding.

RNA-seq from bulk heterogeneous cells, as described in the previous paragraph, fails to resolve the role of specific cell types in the biological system being studied. As HIT pathogenesis is complex and involves multiple cell types, single-cell RNA sequencing (scRNA-seq) may reveal specific roles individual cells play in HIT, and potentially identify cell types that were previously not known to be involved in HIT. ScRNA-seq has been successful in discovering previously unknown cells types and mechanisms involved in phenotypes such as cystic fibrosis and myocardial infarction (Montoro et al., 2018) (Li et al., 2019) (Paik et al., 2020). ScRNA-seq could help answer questions surrounding the individual pathogenic roles of distinct cell types, in the case of monocytes and neutrophils, and the role of cell subtypes, in the case of T-cells and B-cells.

### Proteomics

Proteomic analysis characterizes the proteome, including expression, structure, function, interaction, and modification of proteins (Aslam et al., 2017). While proteomic analysis has brought new insights into HIT, these studies have been limited in scope thus far (Khandelwal et al., 2018). Probing the proteome has the potential to identify a broader range of proteins and post-translational modifications of proteins involved in HIT. Proteomic approaches might be used to determine how glycosylation or post-translational modifications of proteins alters binding affinities and results in differential antibody response. A recent GWAS identified five novel loci associated with IgG antibody glycosylation (Shen et al., 2017).

Although not specific to HIT, proteomic approaches have been used successfully in cohorts of patients treated with heparin and other anticoagulants. One study identified 25 proteins displaying changes in concentration following heparin administration, with 14 proteins replicating in a validation cohort (Beck et al., 2018). In another study of heparin-treated pregnant women, multiplex protein screening identified increased levels of chemokines CXCL10, CLCL11 and CCL20 when compared to untreated pregnant controls (Rasmark Roepke et al., 2019). Another recent study showed substantive changes to expression of focal adhesion and cytoskeletal proteins in podocytes following chronic heparin exposure (Delézay et al., 2021). In a cohort of 4,200 oral anticoagulant patients with atrial fibrillation, one recent study identified seven novel proteins associated with increased risk of bleeding (Siegbahn et al., 2021). A number of other targeted proteomic studies have reported additional biomarkers associated with risk of bleeding in patients with acute coronary syndromes (Hagström et al., 2016; Hijazi et al., 2016). In a study comparing treatment with warfarin and rivaroxaban, nine metaproteins were shown to be differentially expressed (Mueck et al., 2014; Harter et al., 2015; Kaye et al., 2017; Reçber et al., 2020).

Proteomic profiling still suffers from shortcomings, such as the large samples required to achieve an accurate measurement and difficulty accounting for heterogeneity within a sample. Single-cell proteomics is an emerging technique that is applicable to HIT research. Still in its infancy, initial reports of profiling hundreds of proteins from a single, or small number of mammalian cells were first published in 2018 (Budnik et al., 2018; Zhu et al., 2018). As the transcriptome is not a direct reflection of the proteome, and that post-translational modifications of proteins are unattainable from RNA transcripts, the use of proteomics should be strongly considered to probe unanswered questions within the field. Furthermore, proteomics can be a powerful complement to genomic and transcriptomic studies, by offering systems-level insights into HIT pathogenesis.

### Metabolomics

The comprehensive set of small molecules within the body, the metabolome, is another avenue of research for further understanding of HIT pathogenesis. The metabolome can affect biological pathways through the modulation of the genome, transcriptome, and proteome (Rinschen et al., 2019). Metabolites serve as enzyme cofactors and substrates in biochemical processes. Activity of transmembrane receptors and transcription factors can be modulated by metabolites via allosteric regulation and metabolites can influence RNA metabolism by acting on riboswitches (Rinschen et al., 2019).

Metabolites such as physiologically relevant metal cations induce conformational changes in heparin (Seo et al., 2011; Zhang 2014). Neutralization of heparin via metal ion binding could influence the binding of PF4 to heparin since the electrostatic interaction between heparin and PF4 is critical for complex formation. Calcium (Ca^2+^) also induces substantive conformational changes in heparin (Seo et al., 2011) and is a critical cofactor in the binding of heparin to particular proteins such as Annexin A2 (Shao et al., 2006). Changes in concentrations of Ca^2+^ and Zinc (Zn^2+^), alters the binding affinity of heparin to other proteins including fibroblast growth factor-1 and interleukin 7 (Zhang 2014). Zinc (Zn^2+^) is present in high concentrations in *α*-granules, is a critical cofactor in non-HIT thrombosis (Vu et al., 2013), and is known to mediate binding of heparin to proteins including fibrinogen, high molecular weight kininogen, and histidine-rich glycoprotein (Fredenburgh et al., 2013; Sobczak et al., 2018). Substantive changes in the concentrations of these physiologically relevant metal cations could be a risk factor for HITT. Profiling of metal cation concentrations in the serum of HIT cases and heparin-treated controls could identify novel biomarkers in HITT. Metabolites, including acetate, citric acid, 3-hydroxybutarate, glucose, and some mono- and polyunsaturated fatty acids have been shown to be altered in arterial and deep venous thrombosis (Quintero et al., 2020). Metabolomics work could be applied to determine differences in metabolite levels affecting differential antibody responses and differences in HIT risk. The identification of metabolites that are risk factors for HIT could be invaluable within the clinic. Patients with metabolic alterations could alert clinicians to prescribe alternative therapies or increase monitoring of these at-risk patients.

## Conclusion

Heparin-induced thrombocytopenia is a complex, unpredictable, immune-mediated adverse drug reaction involving multiple cell types. While platelets appear to be primarily involved in HIT, strong evidence exists for a critical role of several cell types in HIT pathogenesis. Neutrophils and monocytes have strong evidence for their role in HIT, particularly in HITT. HIT exhibits both T-cell dependent and independent features and genomic studies have observed associations between variation in antigen presentation pathways and HIT. Endothelial cells serve as a deposition site for PF4, likely playing a role in PF4/heparin antibody binding and signal transduction. Despite our understanding of these cell types and their roles in HIT, multiple gaps in our knowledge of HIT pathogenesis remain. Applying multi-omics techniques to HIT has the potential to bring new insights. Larger and functional assay-confirmed HIT cohorts are necessary to overcome the limitations of published HIT genomic studies. Other omics techniques, such as transcriptomics and proteomics, have been underutilized in HIT research. As technologies become more established and less cost prohibitive, single cell transcriptomics and proteomics are particularly well-suited to address for the gaps in our knowledge of HIT pathogenesis. Such omics approaches have great promise to elucidate biological pathways, profile patient samples and discover valuable clinical biomarkers, which could reduce the risk of HIT and improve patient outcomes in the clinic.
